# Stage-Specific Defense Reprogramming in Proso Millet Against Head Smut: Insights from Time-Series Transcriptomic and Metabolomic Integration

**DOI:** 10.3390/biology15171517

**Published:** 2026-09-03

**Authors:** Wenqi Fan, Mingyu Qi, Zhiguang Li, Yanyan Zuo, Jinghan Feng, Min Zhao, Dan Liu, Xiangqi Zhu, Fuli Zhang, Yue Jiang, Liyuan Zhang

**Affiliations:** 1 Chifeng Academy of Agricultural and Animal Husbandry Sciences, Chifeng 024031, China; fanwenqi@alu.cau.edu.cn (W.F.); qimingyu006@126.com (M.Q.); lizg05@126.com (Z.L.); yanyazuo2022@163.com (Y.Z.); fengjh1201@163.com (J.F.); zm0476@163.com (M.Z.); liudy2804@163.com (D.L.); 13664865529@163.com (F.Z.); 15540691511@163.com (Y.J.); 2 Chifeng Agriculture and Animal Husbandry Technology Extension Center, Chifeng 024050, China; cfsnmjstgzx@163.com

**Keywords:** proso millet, smut disease, transcriptomics, multi-omics integration, temporal defense

## Abstract

Smut disease is a major constraint on proso millet (*Panicum miliaceum* L.) production. Our previous metabolomic work in the cultivar ‘Chishu 13’ outlined a two-stage defense strategy involving energy metabolism at jointing and phenylpropanoid activation at heading, yet the transcriptional regulation underlying this metabolic shift remained unknown. In this follow-up study, we performed RNA-seq on the same biological samples used in our prior metabolomics analysis to directly link transcriptomic changes to metabolic outcomes. We compared asymptomatic inoculated (IA) and symptomatic inoculated (IS) plants across four developmental stages (seedling, tillering, jointing, and heading) using time-series profiling, weighted gene co-expression network analysis (WGCNA), and multi-omics integration. Transcriptional reprogramming was minimal during early infection but escalated sharply from jointing (5498 differentially expressed genes) to heading (6818 DEGs), matching the previously observed metabolic divergence. K-means clustering revealed two distinct transcriptional programs: TCA cycle and oxidative phosphorylation pathways dominated at jointing, while phenylpropanoid biosynthesis and plant–pathogen interaction pathways peaked at heading. By combining WGCNA with temporal clustering, we identified 337 high-confidence Stage 1 and 408 Stage 2 candidate genes. Seven hub genes encoding TCA cycle enzymes correlated positively with citrate and succinate accumulation only in IA plants at jointing, whereas eight phenylpropanoid-related hub genes strongly associated with L-phenylalanine and cinnamic acid levels in IA plants at heading. These coordinated transcriptional–metabolic modules were disrupted in IS plants, and RT-qPCR confirmed phenotypic-group-specific expression of key hub genes. This study presents a temporally resolved transcriptional atlas of proso millet response to *Anthracocystis destruens*, extending beyond prior single-time-point multi-omics studies by integrating matched transcriptomic and metabolomic time-series data to establish a dynamic multi-omics framework for smut resistance. We demonstrate that effective defense requires precise sequential coordination of energy and phenylpropanoid metabolism, providing validated hub genes as targets for molecular breeding.

## Simple Summary

Smut disease severely limits proso millet production, yet stage-specific defense mechanisms remain unclear. This study elucidated the dynamic transcriptional basis of resistance by performing time-series RNA-seq on samples from our prior metabolomics work across four developmental stages. Defense reprogramming intensified at jointing and heading, driven sequentially by energy metabolism and phenylpropanoid biosynthesis, respectively. Validated hub genes correlated with key metabolites exclusively in resistant plants, while these modules were disrupted in susceptible ones. We conclude that effective resistance requires precise temporal coordination of these metabolic pathways. This work establishes a temporally resolved transcriptional atlas that complements prior static multi-omics snapshots by capturing dynamic defense reprogramming across four developmental stages and identifies actionable hub gene targets for molecular breeding. These findings support developing resistant cultivars to enhance food security and sustainable agriculture in proso millet-growing regions.

## 1. Introduction

Proso millet (*Panicum miliaceum* L.) is a vital traditional minor cereal crop in the arid and semi-arid regions of northern China, valued for its desirable agronomic traits including drought tolerance, low soil fertility adaptability, and short growth cycle [1,2,3]. Its grains are rich in high-quality protein, dietary fiber, and various trace minerals, conferring significant potential for functional food development. However, production has long been severely constrained by smut disease, caused by the basidiomycete fungus *Anthracocystis destruens* [4,5]. As a typical systemic fungal disease, smut is characterized by asymptomatic colonization following seedling-stage infection, with pathogen development synchronized to that of the host; this ultimately leads to complete panicle destruction and total yield loss at heading. Field incidence typically ranges from 5% to 10% but can exceed 40% under severe epidemics [6,7,8]. Smut also causes extensive damage in related gramineous crops such as maize and sorghum [9]. Given the absence of early diagnostic symptoms and the limited efficacy of chemical control against systemically colonizing hyphae, breeding resistant cultivars remains the most economically viable and environmentally sustainable management strategy [10,11,12].

In recent years, significant progress has been made in elucidating the pathogenic mechanisms and genetic basis of smut resistance in maize and sorghum. For instance, it has been established that the maize smut pathogen invades seedlings via appressoria and systemically colonizes the shoot apical meristem [13], and the key resistance gene ZmWAK, encoding a receptor-like kinase, was cloned through genome-wide association studies and QTL mapping [14]. In sorghum, major QTLs conferring smut resistance have also been mapped using molecular markers [15,16]. Furthermore, *Ustilago maydis* has been shown to manipulate host photosynthesis and senescence to facilitate its own colonization [17], revealing the molecular mechanisms by which pathogens reprogram host metabolism to promote infection [18]. In contrast, research on smut resistance in proso millet remains at a relatively early stage. Although some resistant germplasms have been identified through genetic resource screening [19,20,21], cultivars combining high resistance with stable agronomic performance are still scarce. Early studies primarily focused on field resistance evaluation, fungicide screening, and macroscopic descriptions of the pathogen infection process [22,23,24]. While existing transcriptomic analyses have implicated pathways such as phenylpropanoid biosynthesis, plant–pathogen interactions, calcium signaling, jasmonic acid signaling, and antioxidant systems in the proso millet response to smut [25,26,27,28,29,30], these findings were all derived from single-time-point sampling and thus fail to capture the temporal dynamics of host defense responses during systemic pathogen colonization.

Our previous untargeted metabolomic analysis systematically revealed stage-specific metabolic reprogramming underlying smut resistance in proso millet, identifying two critical defense phases: energy metabolism regulation at jointing and phenylpropanoid pathway activation at heading [31]. However, these dynamic metabolic phenotypes represent only the downstream outcomes of defense responses; the upstream transcriptional regulatory mechanisms remain entirely unknown. The inherent limitations of metabolomics preclude capturing the temporal dynamics of gene expression, identifying key regulators driving metabolic transitions, distinguishing the relative contributions of transcriptional versus post-translational regulation, or establishing causal links between gene expression and metabolite accumulation. Therefore, time-series transcriptomic profiling within the same biological system is urgently needed to bridge this critical knowledge gap.

As a complementary extension of our previous metabolomic study, this work provides a systematic characterization of the temporal transcriptional regulatory network underlying proso millet response to smut infection across the entire disease progression. Unlike prior comparative multi-omics analyses limited to single developmental stages [30], the present study leverages matched transcriptomic and metabolomic samples across four time-points to enable temporal network analysis and delineate stage-specific defense dynamics. Using an identical experimental design and biological samples as in the prior metabolomic analysis, we performed RNA-seq on leaf tissues from inoculated asymptomatic (IA) and inoculated symptomatic (IS) plants at four critical developmental stages: seedling, tillering, jointing, and heading. The key findings of this study are as follows: (1) We constructed the first time-resolved transcriptomic atlas of proso millet in response to smut, systematically delineating the dynamic evolution of transcriptional regulatory networks between resistant and susceptible plants across developmental stages. (2) We identified a series of hub genes and functional modules significantly associated with resistance, encompassing transcription factors, protein kinases, and key metabolic enzymes. (3) Through integrative analysis of transcriptomic and metabolomic data, we established gene–metabolite co-expression networks that elucidate the molecular regulatory mechanisms governing two pivotal defense phases: energy metabolism regulation at jointing and phenylpropanoid pathway activation at heading. (4) We identified multiple candidate master regulators, providing novel targets for dissecting the molecular basis of smut resistance in proso millet. By integrating the transcriptomic data generated here with our prior metabolomic results, we propose a comprehensive “transcription–metabolism” multi-omics regulatory framework. This framework not only uncovers the transcriptional underpinnings of metabolic reprogramming but also reveals the temporal regulatory logic of host defense responses during pathogen colonization, thereby offering both theoretical foundations and candidate gene resources for molecular breeding of smut-resistant cultivars.

## 2. Materials and Methods

### 2.1. Plant Materials, Pathogen Inoculation, and Sampling Strategy

The proso millet cultivar ‘Chishu 13’ was used as the experimental material in this study. The experimental design, pathogen inoculation protocol, and sampling strategy were identical to those described in our previous metabolomic study [31]. Briefly, seeds were artificially inoculated with teliospores of *A. destruens* via dry seed coating at a mass ratio of 1:350 (teliospores:seeds) and sown in a controlled-environment chamber maintained at 28 °C, 80% relative humidity, and a 16-h photoperiod. After uniform seedling emergence, plants were transplanted to an experimental field and managed under standard agronomic practices.

**Figure 1:**
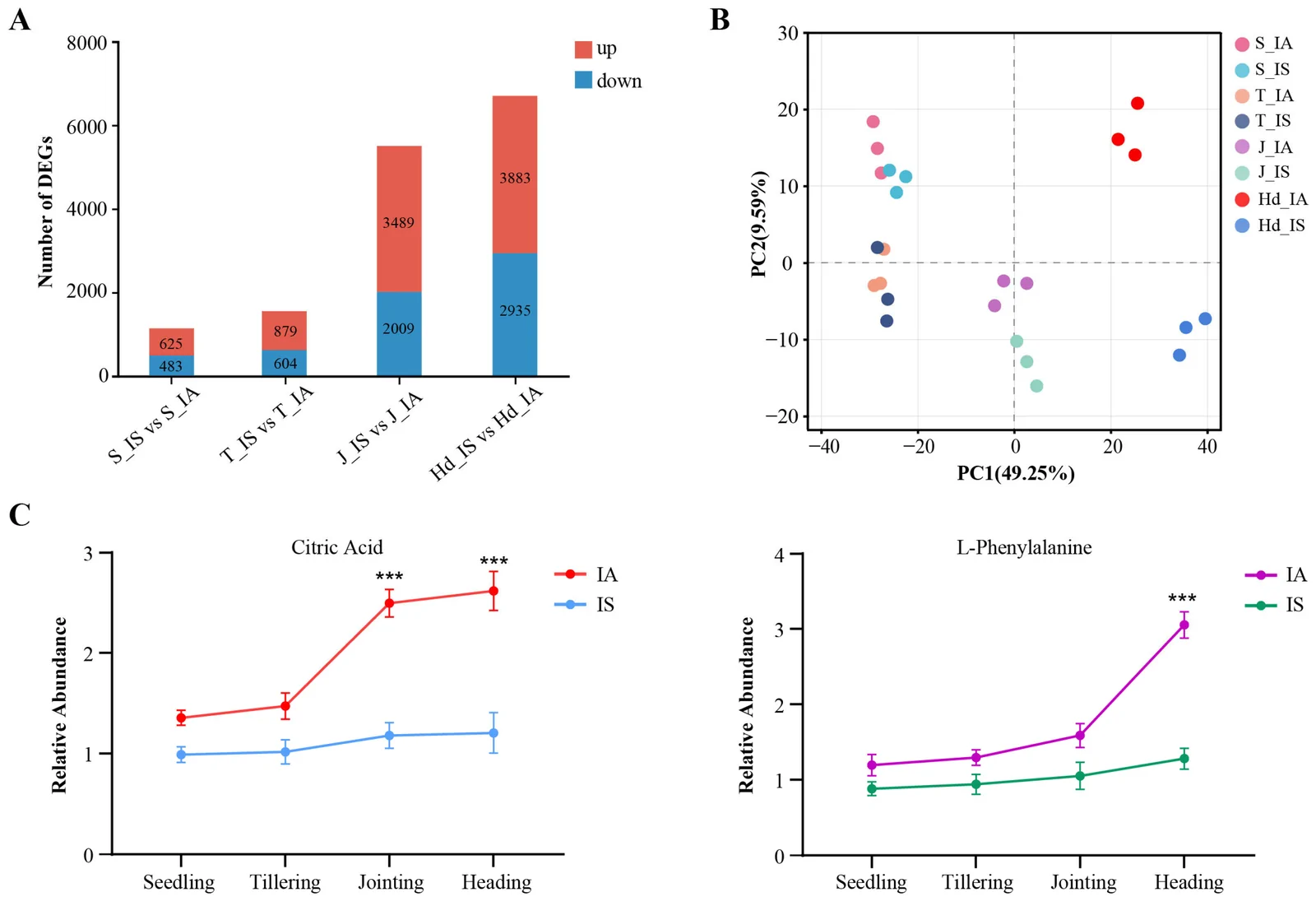
Temporal transcriptomic divergence between IA and IS proso millet during A. destruens infection. (**A**) The number of DEGs across the four developmental stages (S, seedling; T, tillering; J, jointing; Hd, heading). (**B**) PCA showing sample separation starting from the jointing stage. (**C**) Metabolomic profiles adapted from ref. [31] with permission. Temporal expression dynamics of two key metabolites (citrate and L-phenylalanine) in the metabolomic data. Statistical significance was defined as *p* < 0.05 (***).

A total of 200 individual plants were cultivated, and functional leaf samples were collected at four key developmental stages: seedling, tillering, jointing, and heading. At the heading stage, plants were retrospectively classified into two groups based on symptom expression: the inoculated asymptomatic (IA) group, comprising plants that remained symptom-free after inoculation, and the inoculated symptomatic (IS) group, comprising plants that developed visible smut symptoms. Six phenotypically stable and representative individuals were randomly selected from each group, yielding a total of 48 samples (6 individuals × 2 phenotypic groups × 4 time-points) for subsequent transcriptomic analysis. The biological samples used in this study were identical to those employed in the previous metabolomic analysis, ensuring direct comparability between transcriptomic and metabolomic datasets. The metabolomic data presented in Figure 1C,D were generated and published in our previous work [31].

### 2.2. Transcriptome Sequencing and Data Preprocessing

Total RNA was extracted using the EASYspin Plant Total RNA Rapid Extraction Kit (Biomed Company, Beijing, China) according to the manufacturer’s instructions. RNA quality and quantity were assessed using a NanoDrop 2000 spectrophotometer (Thermo Fisher Scientific, Waltham, MA, USA) and an Agilent 2100 Bioanalyzer (Agilent Technologies, Santa Clara, CA, USA). Samples meeting predefined quality criteria were selected for library construction.

RNA sequencing was performed on the Illumina HiSeq 4000 platform (Illumina, San Diego, CA, USA) at Shanghai Majorbio Bio-Pharm Technology Co., Ltd. (Shanghai, China), following standard library preparation and sequencing protocols. Raw sequencing reads were processed using Trimmomatic v0.39 to remove adapter sequences and low-quality bases, and short reads were discarded. The resulting clean reads were aligned to the proso millet reference genome (GCA_003046395.2) using HISAT2 v2.2.1, and gene expression levels were quantified as FPKM values using StringTie v2.2.1 [32]. Data reliability among biological replicates was evaluated by Pearson correlation analysis.

### 2.3. Transcriptomic Data Analysis

Differential expression analysis was performed using DESeq2 (v1.36.0) to identify differentially expressed genes (DEGs). PCA was conducted to evaluate sample clustering, and volcano plots and heatmaps were generated using ggplot2 (v3.5.1) and pheatmap (v1.0.13) to visualize DEGs. Functional enrichment analysis of DEGs was carried out using clusterProfiler (v4.4.0) for GO terms and the KOBAS (v3.0) server for KEGG pathways, with significance defined as FDR < 0.05. To further dissect the temporal dynamics of gene expression, soft clustering of DEGs was performed using the Mfuzz package (v2.56.0); the optimal number of clusters was determined via the silhouette coefficient method, followed by functional enrichment analysis for each cluster to identify stage-specific biological processes. Additionally, WGCNA was performed using the WGCNA package (v1.71). Genes were filtered prior to network construction, and module–trait correlations and hub genes were identified based on predefined statistical criteria.

### 2.4. RT-qPCR Validation

To validate the RNA-seq results, RT-qPCR was performed using the SYBR Green I Master Kit (Tiangen Biotech Co., Ltd., Beijing, China) according to the manufacturer’s instructions. Gene-specific primers were designed using Primer Express v3.0 (Table S2). The proso millet Actin gene was used as an internal reference, and relative expression levels were calculated using the 2^−ΔΔCt^ method. RT-qPCR was conducted on a StepOnePlus Real-Time PCR System (Thermo Fisher Scientific, Waltham, MA, USA) in 96-well plates with the following thermal cycling protocol: initial denaturation at 95 °C for 10 min, followed by 40 cycles of 95 °C for 15 s and 60 °C for 32 s, and a final melt curve stage consisting of 95 °C for 15 s, 60 °C for 1 min, and 95 °C for 15 s.

### 2.5. Statistical Analysis

All statistical thresholds and quality control criteria applied in this study are defined as follows: For RNA-seq library construction, only samples with RNA integrity number (RIN) ≥ 8.0, A260/A280 ratio of 1.8–2.0, and A260/A230 ratio > 2.0 were retained. During read preprocessing, bases with Q < 20 were trimmed, and reads shorter than 50 bp were discarded. Data reliability was ensured by requiring Pearson correlation coefficients ≥ 0.9 among biological replicates. DEGs were identified using |log_2_(fold change)| ≥ 1 and false discovery rate (FDR) < 0.05. Functional enrichment results (GO and KEGG) were considered significant at FDR < 0.05. In WGCNA, genes with FPKM ≥ 1 in at least 80% of samples were retained; the soft-thresholding power was set to 12 to achieve a scale-free topology fit index (R^2^) of 0.9, and the minimum module size was 30 genes. Module–trait correlations were deemed statistically significant when |r| > 0.7 and *p* < 0.01. Hub genes were defined by module membership (kME > 0.95) and gene significance (GS > 0.2). ChatGPT-3.5 was used solely for language refinement and verification of this manuscript.

## 3. Results

### 3.1. Transcriptional Reprogramming During A. destruens Infection in Proso Millet

Our previous metabolomic study established a two-stage defense model against *A. destruens* in the proso millet cultivar ‘Chishu 13’. To elucidate the underlying transcriptional regulatory mechanisms, we performed RNA-seq on samples from an identical experimental design. Leaf tissues were collected from IA and IS plants at four key developmental stages: seedling, tillering, jointing, and heading. These time-points were selected based on our prior finding that metabolic divergence initiates at jointing and peaks at heading. The use of matched biological samples enabled direct integration of transcriptomic and metabolomic datasets to comprehensively characterize the molecular defense responses.

Comparative analysis revealed limited transcriptomic divergence at early developmental stages, with 1108 and 1483 differentially expressed genes (DEGs) identified at seedling and tillering, respectively (Figure 1A). In contrast, jointing and heading exhibited substantial transcriptional reprogramming, with 5498 and 6818 DEGs, respectively. This sharp increase in DEG abundance at later stages indicates that the transcriptional response of proso millet to *A. destruens* is developmentally regulated. Although IA and IS samples clustered together at seedling and tillering, they separated distinctly along PC1 at jointing, with further divergence at heading (Figure 1B). As previously reported in our metabolomics study [31], the proso millet cultivar ‘Chishu 13’ exhibits a two-stage metabolic defense strategy against *A. destruens*, characterized by TCA cycle intermediate accumulation at jointing and phenylpropanoid pathway activation at heading. These established metabolic phenotypes provide the essential biological context and validation foundation for the present transcriptomic analysis. Integration with these previously published metabolite profiling data revealed coordinated multi-omics changes: at jointing, elevated citrate levels in IA plants coincided with transcriptional activation of tricarboxylic acid (TCA) cycle genes, while peak phenylalanine accumulation at heading corresponded to upregulation of phenylpropanoid biosynthesis genes (Figure 1C). These temporally aligned transcriptomic and metabolomic shifts support a sequential defense strategy in which smut resistance in proso millet involves the ordered activation of energy metabolism followed by secondary metabolite biosynthesis.

### 3.2. Temporal Dynamics of Differentially Expressed Genes

**Figure 2:**
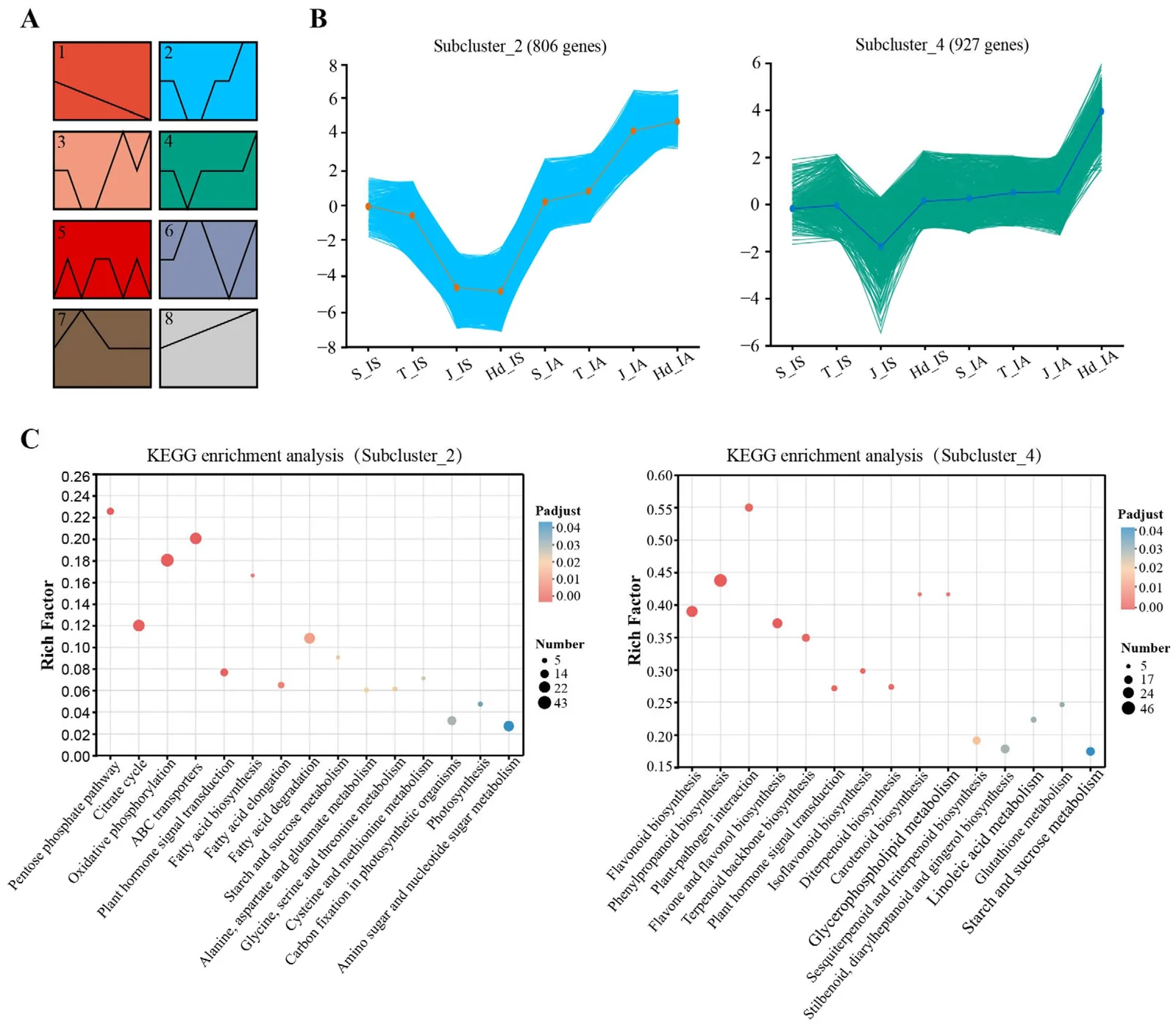
K-means clustering reveals stage-specific transcriptional programs underlying the two-stage defense model. (**A**) Eight distinct expression subclusters identified by temporal K-means clustering. The x-axis represents the four developmental stages (seedling, tillering, jointing, and heading) in both IS and IA plants, and the y-axis represents normalized gene expression levels (Z-score). Eight distinct expression subclusters identified by temporal K-means clustering. (**B**) Detailed temporal expression profiles of Subcluster 2 and Subcluster 4. (**C**) KEGG enrichment analysis of Subcluster 2 (**left**) and Subcluster 4 (**right**).

K-means clustering based on time-series expression patterns partitioned the DEGs into eight distinct subclusters (Figure 2A). Among these, Subcluster 2 (806 genes) exhibited jointing-specific upregulation exclusively in IA plants, while Subcluster 4 showed heading-specific induction predominantly in the IA group with concurrent suppression in IS plants. These two subclusters were selected for detailed characterization (Figure 2B) because their opposing expression trends between IA and IS plants—upregulation in resistant versus downregulation in susceptible individuals—closely mirrored the stage-specific accumulation patterns of TCA cycle intermediates and phenylpropanoid metabolites observed in our prior metabolomic profiling [31], providing direct transcriptomic evidence supporting the two-stage defense model. The remaining six subclusters lacked such consistent multi-omics concordance and were therefore not pursued further.

To elucidate the functional relevance of these stage-specific DEGs, we performed KEGG pathway enrichment analysis. Genes in Subcluster 2 were significantly enriched in central metabolic pathways, including the TCA cycle, oxidative phosphorylation, ABC transporters, and the pentose phosphate pathway (Figure 2C). In contrast, genes in Subcluster 4 were predominantly enriched in secondary metabolism and defense-related pathways, including flavonoid biosynthesis, phenylpropanoid biosynthesis, plant–pathogen interaction, and flavone and flavonol biosynthesis (Figure 2C).

### 3.3. WGCNA Identifies Stage-Specific Regulatory Modules Associated with Resistance in IA Plants

**Figure 3:**
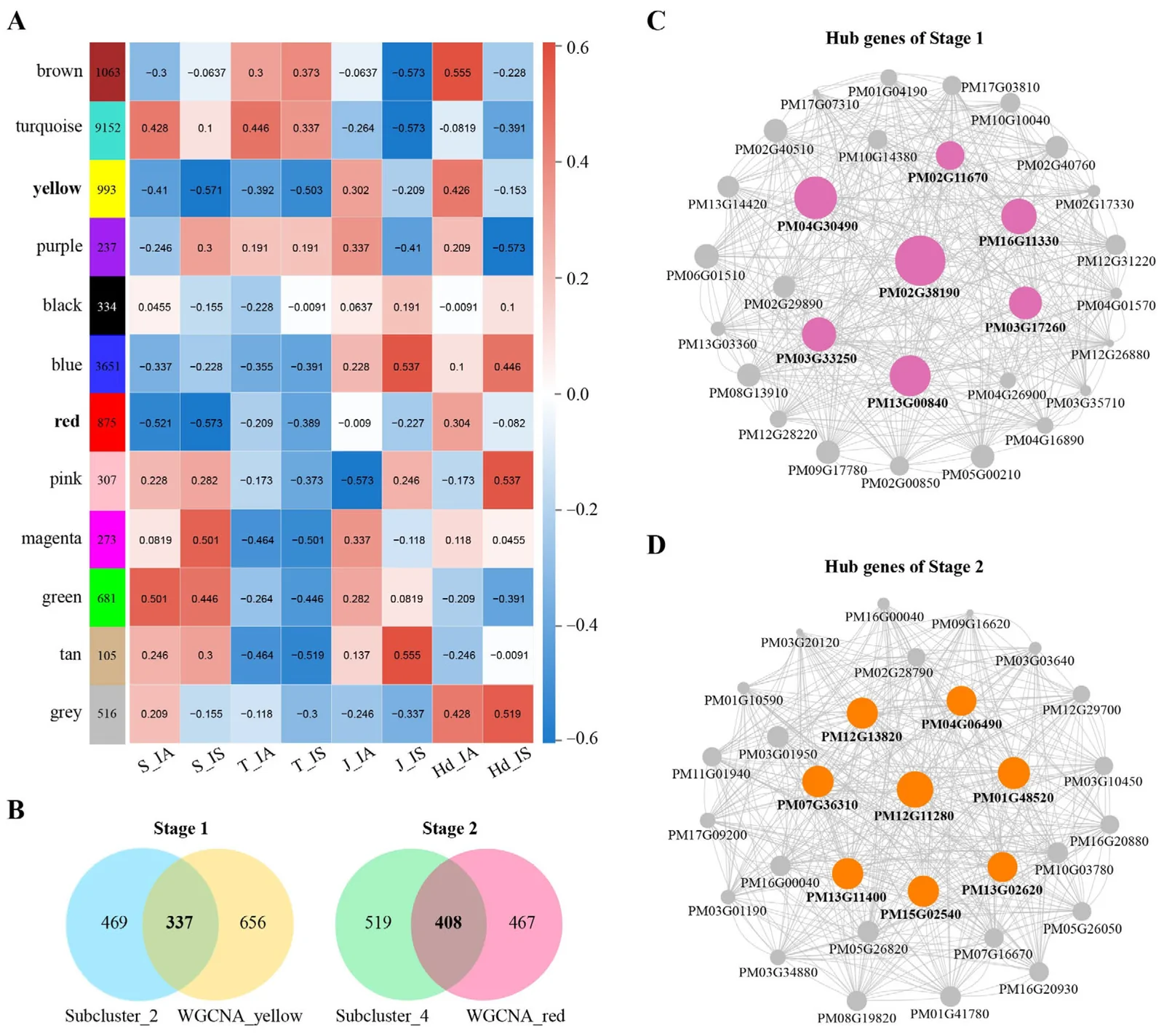
WGCNA identifies stage-specific co-expression modules associated with resistance. (**A**) Genome-wide module–trait association heatmap analysis. (**B**) Venn diagrams yielding 337 Stage 1 and 408 Stage 2 high-confidence candidate genes. (**C**) Topological network of top 30 hub genes highlighting core hubs with kME > 0.95 in Stage 1. (**D**) Topological network of top 30 hub genes highlighting core hubs with kME > 0.95 in Stage 2.

To systematically identify genome-wide regulatory modules associated with disease resistance, we performed weighted gene co-expression network analysis (WGCNA) on all DEGs, which yielded 12 distinct co-expression modules (Figure 3A). The yellow module was positively correlated with IA plants at jointing, while the red module showed a positive correlation with IA plants at heading.

To obtain high-confidence candidate genes, we intersected the subclusters identified by TSTA with the modules defined by WGCNA. Specifically, the intersection of Subcluster 2 (jointing-specific) and the yellow module yielded 337 candidates specific to the jointing stage (Stage 1), while the intersection of Subcluster 4 (heading-specific) and the red module yielded 408 candidates specific to the heading stage (Stage 2) (Figure 3B). These genes exhibit both temporal specificity and strong co-expression patterns. Topological analysis of the top 30 hub genes within each stage revealed highly interconnected co-expression network structures in both Stage 1 (jointing) (Figure 3C) and Stage 2 (heading) (Figure 3D). Notably, seven hub genes in Stage 1 and eight in Stage 2 displayed module eigengene connectivity (kME) values exceeding 0.95, indicating their central positions within their respective regulatory networks.

### 3.4. Population-Specific Association Between Stage 1 Hub Genes and TCA Cycle Metabolites

Our previous metabolomic analysis revealed that TCA cycle metabolites, including citrate, succinate, and fumarate, are significantly enriched during Stage 1 (jointing) of the defense response, representing key metabolic features of this stage. To investigate the relationship between gene expression and metabolite abundance, we performed correlation analysis between the top 30 hub genes identified in Stage 1 and these three TCA cycle metabolites.

**Figure 4:**
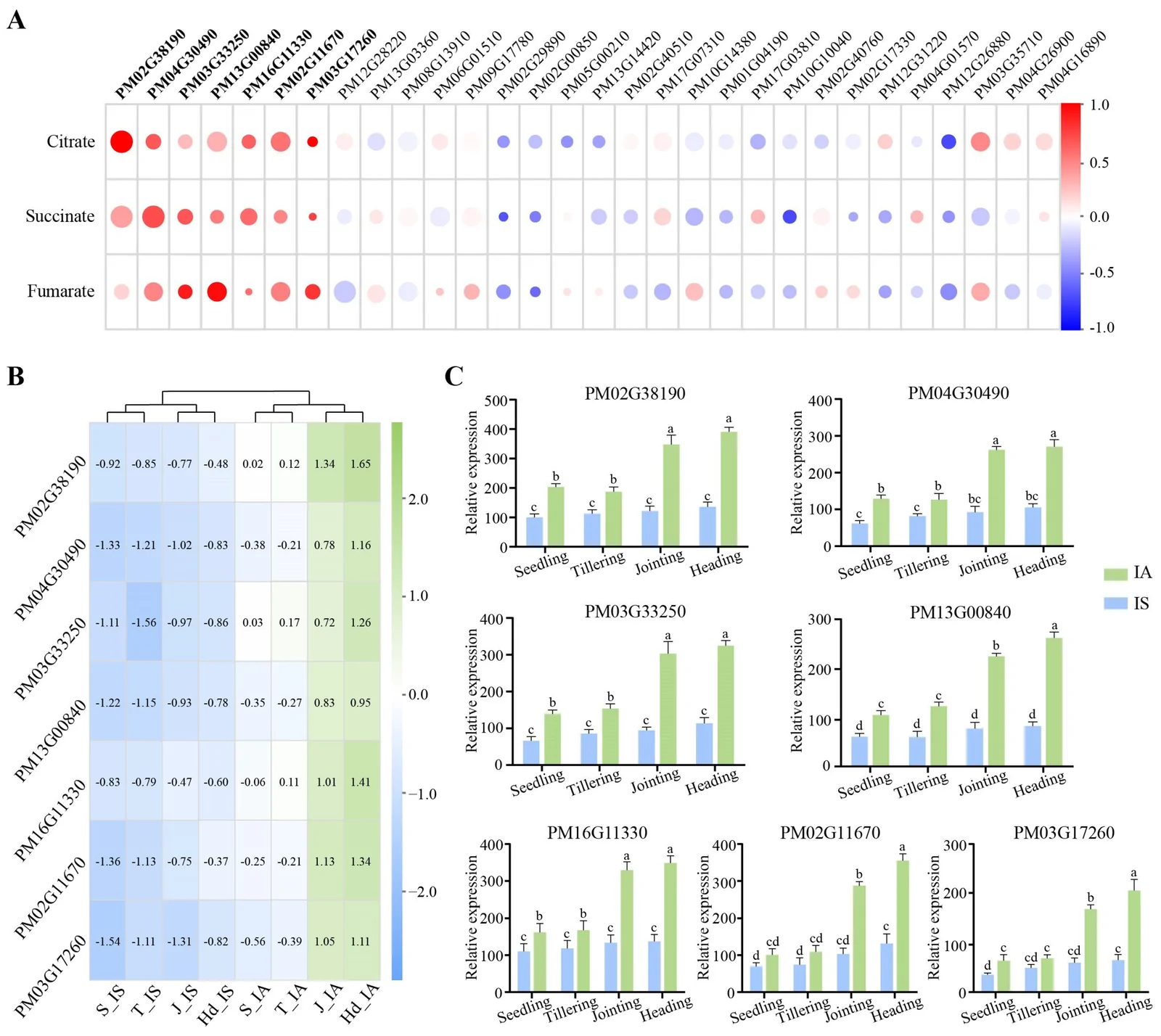
Phenotypic-group-specific association of Stage 1 hub genes with TCA cycle metabolites at jointing. (**A**) Pearson correlation identifying seven hub genes significantly correlated with three TCA metabolites in IA plants. (**B**) Temporal expression heatmap showing coordinated upregulation exclusively in IA plants. (**C**) RT-qPCR validation confirming phenotypic-group-specific expression patterns consistent with RNA-seq. Data are mean ± SD (n = 3 biological replicates). Different lowercase letters indicate significant differences (one-way ANOVA with Tukey’s HSD, *p* < 0.05).

In the IA population, expression levels of seven hub genes (PM02G38190, PM04G30490, PM03G33250, PM13G00840, PM16G11330, PM02G11670, and PM03G17260) were positively correlated with TCA cycle metabolite abundance (Figure 4A), indicating a coordinated transcriptional–metabolic regulatory network in IA plants at jointing. Temporal expression heatmaps further showed that these seven hub genes exhibited highly synchronized upregulation in the IA population at jointing, whereas their expression was markedly reduced in the IS population at the same stage (Figure 4B). qPCR validation results were highly consistent with RNA-seq data, confirming significant differential expression of these seven hub genes between IA and IS populations (Figure 4C). Collectively, these findings suggest that the seven Stage 1 hub genes may provide the metabolic foundation for early-stage defense in resistant plants by modulating TCA cycle flux.

### 3.5. Stage 2-Specific Transcriptional Regulation of the Phenylpropanoid Pathway

Metabolomic profiling identified nine phenylpropanoid pathway intermediates—L-phenylalanine, cinnamic acid, cinnamaldehyde, p-coumaric acid, p-coumaraldehyde, phenethylamine, phenylacetaldehyde, phenylacetate, and phenylacetylglycine—that were significantly enriched during Stage 2 of the defense response. To elucidate the transcriptional regulatory mechanisms underlying this metabolic shift, we performed correlation analysis between the top 30 hub genes identified in Stage 2 and the abundance of these nine phenylpropanoid metabolites.

**Figure 5:**
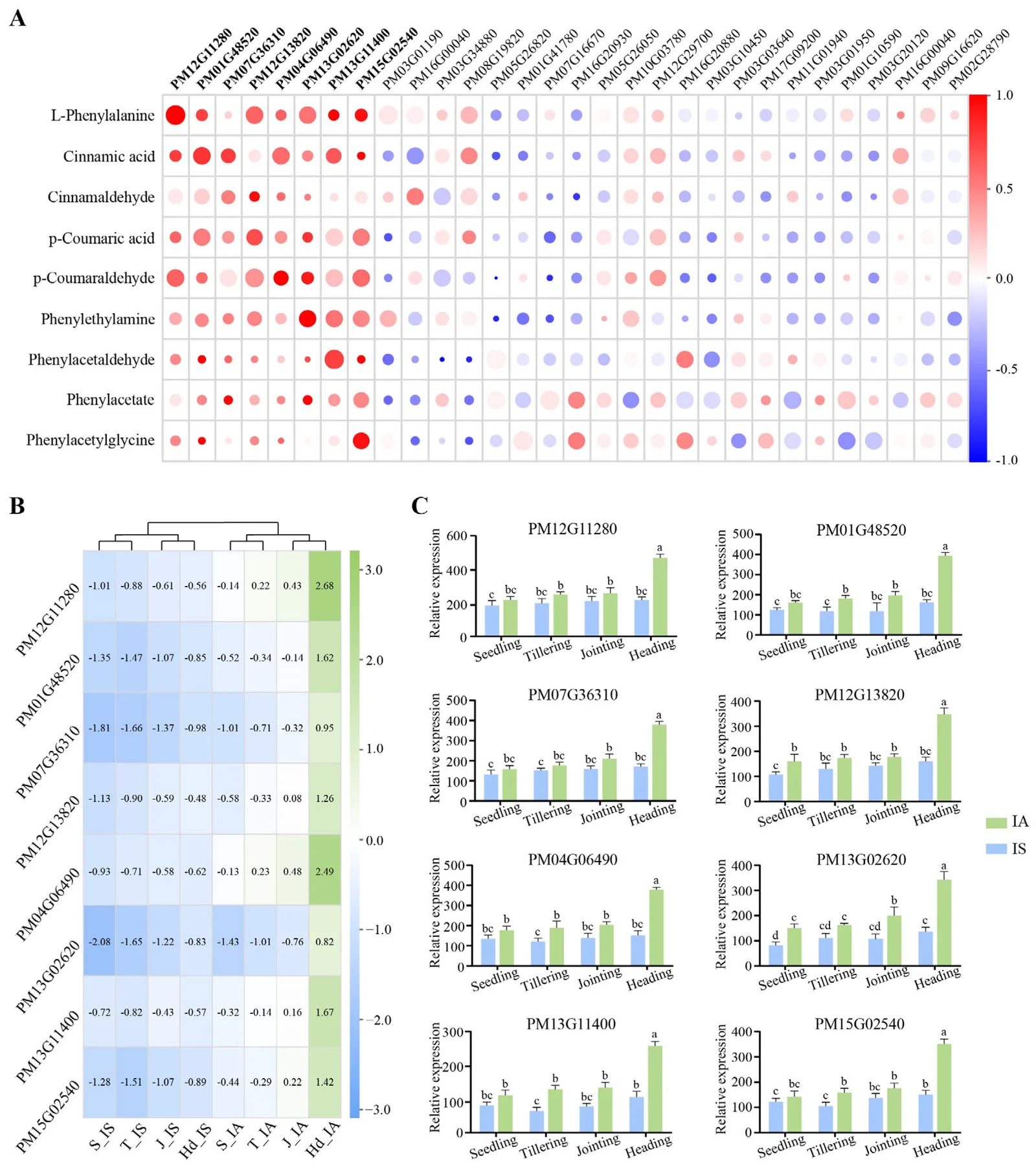
Transcriptional coordination of Stage 2 hub genes with phenylpropanoid accumulation at heading. (**A**) Correlation network between eight hub genes and nine phenylpropanoid metabolites in IA plants. (**B**) Temporal profiles revealing synchronized upregulation in IA versus suppressed expression in IS plants. (**C**) RT-qPCR verification corroborating phenotypic-group-specific transcriptional reprogramming. Data are mean ± SD (n = 3 biological replicates). Different lowercase letters indicate significant differences (one-way ANOVA with Tukey’s HSD, *p* < 0.05).

Pearson correlation analysis revealed that, in IA plants, expression levels of eight hub genes (PM12G11280, PM01G48520, PM07G36310, PM12G13820, PM04G06490, PM13G02620, PM13G11400, and PM15G02540) were positively correlated with phenylpropanoid metabolite accumulation, indicating coordinated transcriptional regulation of this pathway in the resistant phenotypic group (Figure 5A). Temporal expression profiling further demonstrated that these eight hub genes formed a tightly co-regulated transcriptional module in IA plants at heading, with all genes exhibiting synchronous upregulation. In contrast, this transcriptional module was largely suppressed in IS plants at the same developmental stage, displaying heterogeneous and reduced expression patterns (Figure 5B). Quantitative real-time PCR validation confirmed the RNA-seq results, showing high consistency between the two platforms and corroborating phenotypic-group-specific transcriptional reprogramming of phenylpropanoid pathway genes during the Stage 2 defense response (Figure 5C).

### 3.6. Coordinated Transcriptional–Metabolic Regulation Underlying Two-Stage Metabolic Reprogramming

**Figure 6:**
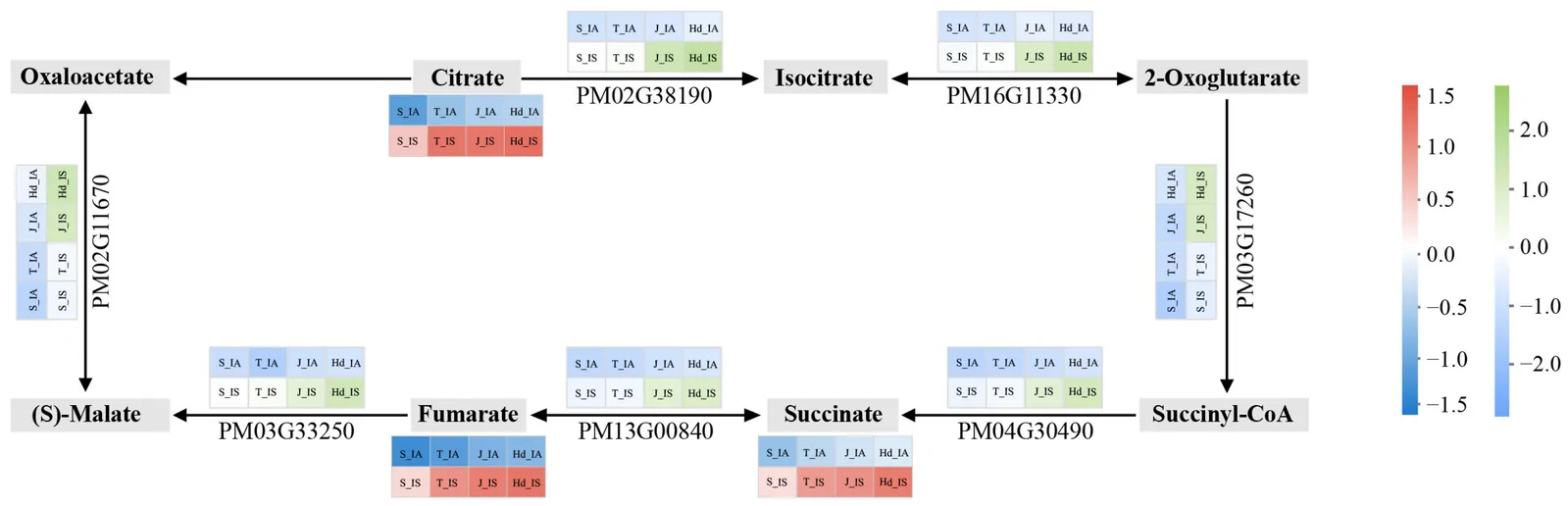
Stage 1 defense mechanism: TCA cycle-driven metabolic reprogramming at the jointing stage.

Transcriptome–metabolome association network analysis identified seven candidate genes significantly correlated with TCA cycle metabolites; these genes encode key enzymes including ACO, IDH1, and LSC1 that catalyze rate-limiting reactions in the TCA cycle (Table S1). Transcript levels of these genes showed coordinated variation with the accumulation of their corresponding metabolites. At jointing, these genes exhibited elevated expression in the IA population but were suppressed in the IS population (Figure 6). This phenotypic-group-specific expression pattern establishes an efficient primary metabolic regulatory module that accelerates carbon skeleton turnover and provides sufficient metabolic precursors and energy reserves for the subsequent activation of secondary metabolic networks.

**Figure 7:**
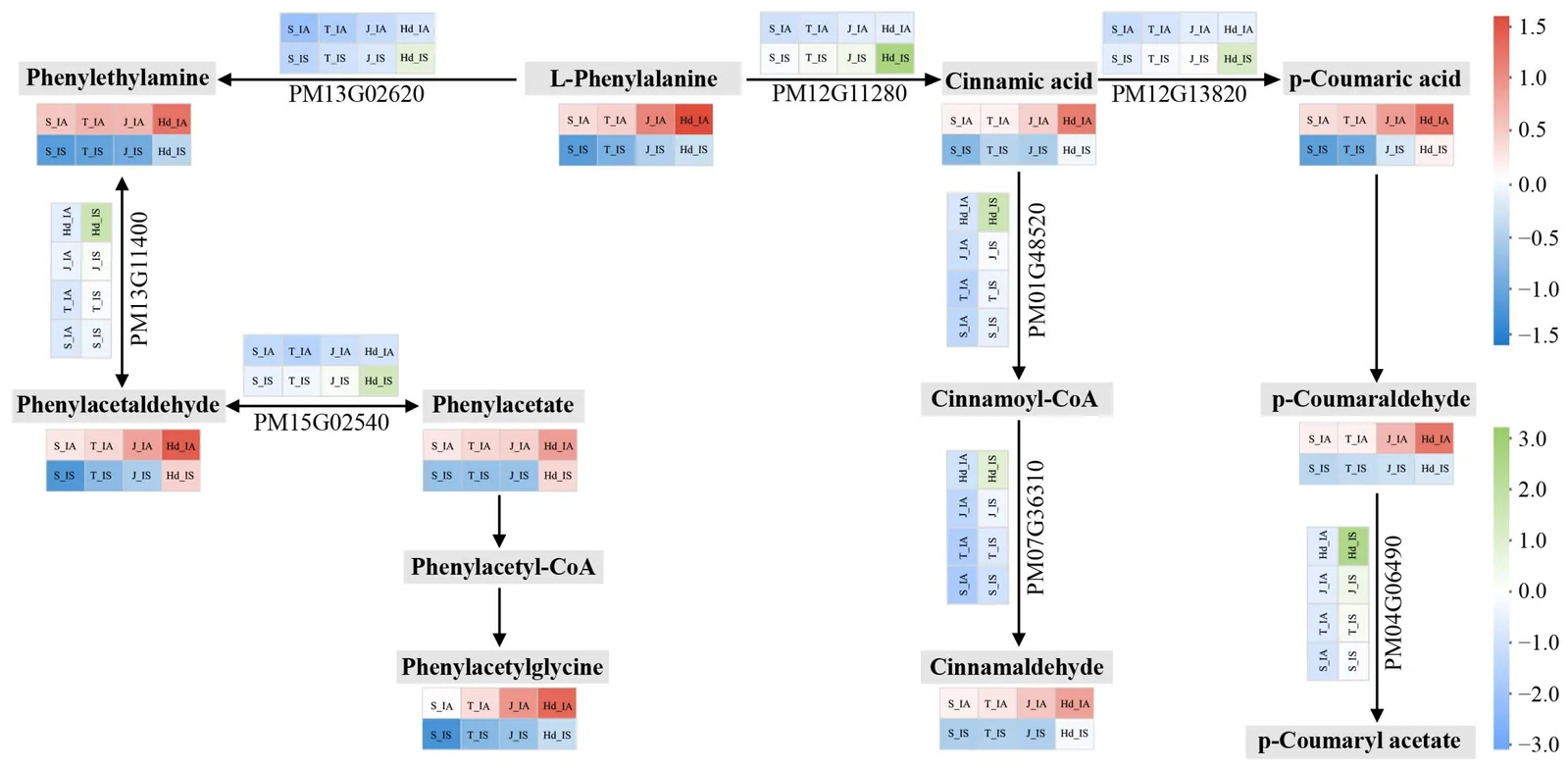
Stage 2 defense mechanism: phenylpropanoid biosynthesis-mediated defense execution at the heading stage.

In Stage 2, eight candidate key genes involved in phenylpropanoid pathway regulation encode rate-limiting enzymes including PCL, 4CL, and CAD, which catalyze the conversion from phenylalanine to downstream metabolites (Table S1). At heading, expression levels of these genes were markedly higher in the IA population than in the IS population, showing clear phenotypic-group-specific differentiation (Figure 7). This differential expression pattern indicates that the phenylpropanoid pathway forms a functionally complete secondary metabolic regulatory unit in the resistant phenotypic group, providing the molecular basis for efficient synthesis of plant defense compounds.

## 4. Discussion

PCA revealed that the transcriptomes of IA and IS plants were highly overlapping during seedling and tillering stages, with divergence emerging only at jointing (Figure 1). This pattern aligns closely with the latent–explosive infection strategy of *A. destruens* as a systemic pathogen. During vegetative growth, the pathogen persists at minimal biomass within vascular tissues, insufficient to trigger basal immunity mediated by host pattern recognition receptors; phenotypic-group-specific transcriptional reprogramming in the resistant line is activated only when the host transitions to reproductive growth and the pathogen synchronously initiates rapid proliferation. Although IS plants also generated substantial numbers of DEGs at jointing and heading (Figure 2), functional enrichment results exhibited high dispersion (Figure 3) and lacked correspondence with metabolite fluctuations observed in metabolomic profiling [31]. These findings indicate that transcriptional changes in susceptible plants more likely represent passive reflections of cellular homeostasis disruption driven by pathogen effector interference, rather than actively orchestrated defense programs. Thus, resistance hinges not on whether a transcriptional response is elicited, but on whether such responses are activated within the correct temporal window as coordinated regulatory modules. Importantly, this temporal resolution distinguishes our work from prior single-stage multi-omics studies [30], which captured only static snapshots insufficient to reveal sequential coordination of defense reprogramming. By integrating matched time-series data across four stages, we uncover stage-specific regulatory hubs and dynamic network rewiring invisible to single-time-point designs, providing a mechanistically richer foundation for translating omics findings into breeding strategies.

The IA-specific co-expression module at jointing identified by WGCNA (Figure 4A) is central to this study; its hub genes predominantly encode TCA cycle enzymes and oxidative phosphorylation complex subunits (Figure 5A), which fully corresponds to the accumulation of intermediates including citrate and succinate in IA plants at the same stage observed in our previous metabolomic analysis [31]. Jointing represents a critical source–sink transition point in proso millet and marks the onset of pathogen shift from latent to active colonization. The coordinated upregulation of respiratory metabolism genes at this stage likely serves three concurrent purposes: reserving ATP and NADPH for subsequent secondary metabolite biosynthesis, supplying carbon skeletons as precursors for the phenylpropanoid pathway, and maintaining mitochondrial electron transport efficiency to prevent self-damage caused by excessive ROS accumulation. IS plants failed to activate this module at the same stage (Figure 4B); TCA cycle gene expression was even lower than that of controls, consistent with reduced energy metabolite levels in metabolomic profiling. This deficiency may impair effective initiation of downstream defense responses due to insufficient energy and precursor supply, constituting the first critical node in resistance–susceptibility differentiation.

Following the energy preparation phase at jointing, the IA-specific co-expression module for phenylpropanoid metabolism activated at heading (Figure 4C) represents the execution stage of defense. Its hub genes encode key enzymes in phenylpropanoid/flavonoid biosynthesis, including PAL, C4H, 4CL, and CHS (Figure 5B), and their expression dynamics are significantly positively correlated with the burst accumulation of phenolic acids and flavonoids observed in metabolomic profiling. This temporal concordance between transcript and metabolite levels suggests that regulation of this pathway occurs primarily at the transcriptional level. Phenylpropanoid derivatives may contribute to disease resistance through multiple mechanisms: lignin deposition reinforcing physical barriers in vascular tissues, phenolic compounds directly inhibiting pathogen growth, and flavonoids acting as signaling molecules to mediate systemic resistance. Although partial upregulation of pathway genes was detected in IS plants at heading (Figure 3D), the magnitude of induction was markedly lower than that in IA plants and showed no quantitative association with metabolite accumulation (Figure 7). These findings indicate that IS plants retain the potential for pathway activation but fail to achieve effective metabolic output due to upstream regulatory defects or pathogen interference.

**Figure 8:**
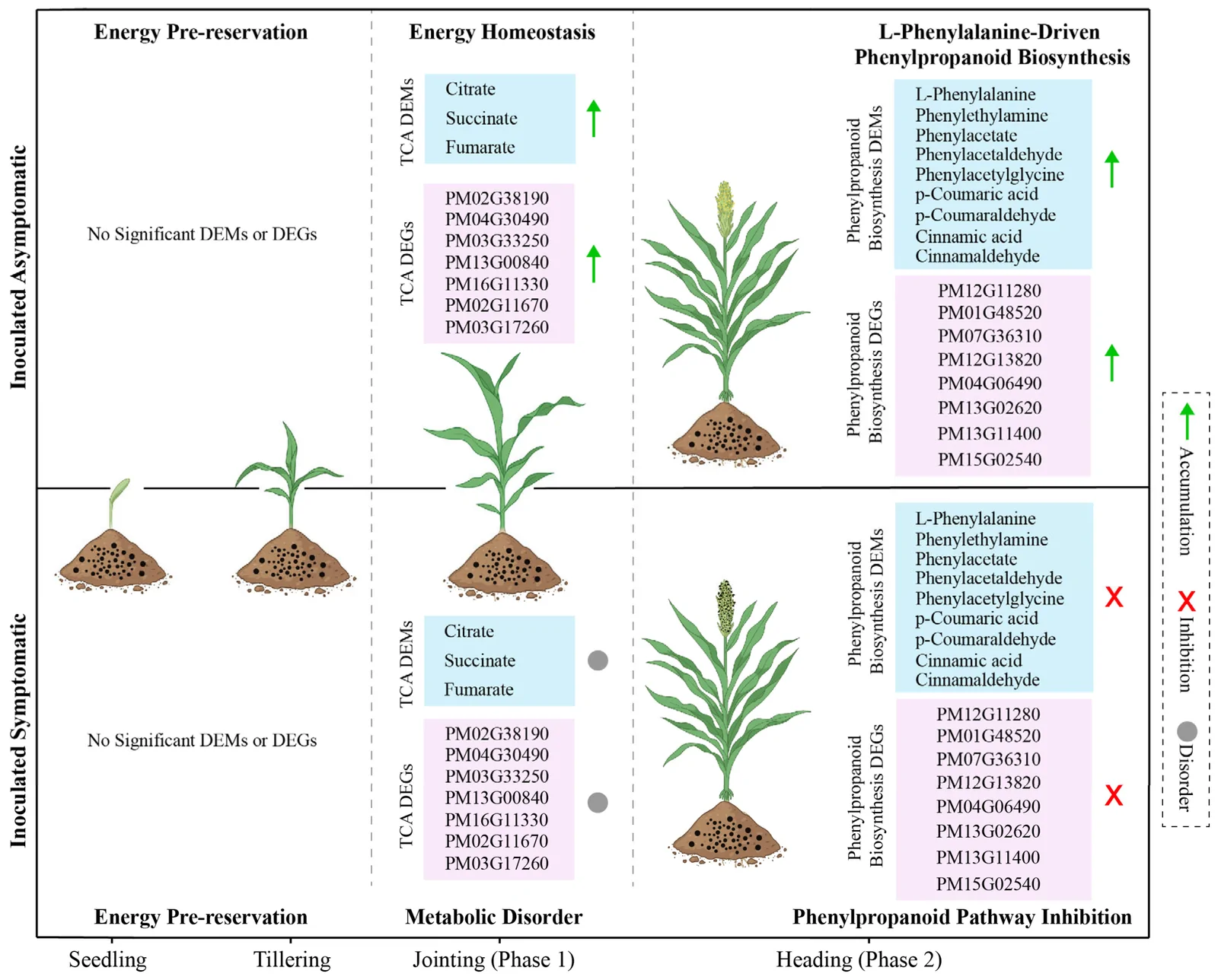
Schematic representation of the two-phase metabolic response model in IA versus IS proso millet during *A. destruens* challenge. The schematic was created in BioRender https://BioRender.com/qug4teh (accessed on 26 February 2026).

Cross-omics integration analysis (Figure 8) supports a two-stage serial regulatory model: respiratory metabolic reprogramming at jointing provides the material and energetic foundation for phenylpropanoid-mediated defense at heading, with the two stages linked by metabolic–signaling feedback loops. Our previous metabolomic profiling confirmed significant accumulation of TCA cycle intermediates (citrate and succinate) in IA plants at jointing; these metabolites serve not only as central nodes in energy metabolism but also as carbon skeleton donors and reducing power suppliers, directly influencing flux partitioning in the downstream phenylpropanoid pathway. The strong co-expression between seven TCA hub genes and eight phenylpropanoid hub genes observed in IA plants (Figure 6) was completely decoupled in IS plants (Figure 7), further confirming that the integrity of this cascade is a prerequisite for the resistant phenotype. This model provides a testable hypothesis for understanding resistance to systemic fungal diseases in gramineous crops: resistance depends on the coordinated activation of multiple physiological modules within precise temporal windows, rather than the mere presence or absence of a single defense pathway.

Building on this mechanistic framework, translating this two-stage transcriptional–metabolic framework into sustainable smut-resistant breeding requires moving beyond sterile, single-organism models toward a holobiont perspective. A key priority is determining whether stress-induced respiratory reprogramming can be enhanced through targeted manipulation of TCA cycle enzymes (e.g., ACO, IDH) or mitochondrial uncoupling proteins, though such efforts must be validated under *A. destruens* challenge to account for potential metabolic suppression by pathogen effectors. Equally critical is the role of endophytes, which remain largely absent from current models; gnotobiotic and synthetic community assays could reveal whether specific taxa prime TCA flux at jointing or potentiate phenylpropanoid responses at heading and whether resistant phenotypic groups actively recruit beneficial microbiota as part of their defense strategy. Ultimately, biomarker discovery (e.g., hub genes PM02G38190, PM12G11280) should prioritize robustness across variable soil microbiomes and field conditions rather than performance in isolation, ensuring next-generation cultivars are resilient within the complex host–microbe–environment interactions that define sustainable agroecosystems.

## 5. Conclusions

This study provides a systematic characterization of the temporal transcriptional regulatory network underlying proso millet (*Panicum miliaceum* L.) response to smut infection, advancing beyond prior static multi-omics comparisons through time-series integration. By integrating longitudinally matched transcriptomic and metabolomic datasets, we elucidated the transcriptional basis of the previously identified two-stage defense model characterized by early energy metabolism reprogramming followed by late phenylpropanoid pathway activation, revealing the dynamic evolution of transcriptional networks across developmental stages in resistant and susceptible plants. We identified key hub genes, functional modules, and candidate master regulators and constructed gene–metabolite co-expression networks to decipher the molecular mechanisms governing the coordinated regulation of energy and secondary metabolism during these two distinct phases. Collectively, we propose a comprehensive “transcription–metabolism” multi-omics regulatory framework, providing a theoretical foundation and candidate genetic resources for the molecular design breeding of smut-resistant proso millet cultivars.

## Data Availability

The original contributions presented in this study are included in the article and Supplementary Materials. The raw transcriptome sequencing data have been deposited in the NCBI SRA database (Accession No. PRJNA1513485).

## Supplementary Materials

The following supporting information can be downloaded at https://www.mdpi.com/article/10.3390/biology15171517/s1: Table S1: Annotation of hub genes; Table S2: RT-qPCR primers.

## References

[1] Lu H., Zhang J., Liu K.-b., Wu N., Li Y., Zhou K. (2009). Earliest domestication of common millet (*Panicum miliaceum*) in East Asia extended to 10,000 years ago. Proc. Natl. Acad. Sci. USA.

[2] Hunt H.V., Badakshi F., Romanova O., Howe C.J., Jones M.K., Heslop-Harrison J.S.P. (2014). Reticulate evolution in Panicum (Poaceae): The origin of tetraploid broomcorn millet, *P. Miliaceum*. J. Exp. Bot..

[3] Liu M., Xu Y., He J., Zhang S., Wang Y., Lu P. (2016). Genetic Diversity and Population Structure of Broomcorn Millet (*Panicum miliaceum* L.) Cultivars and Landraces in China Based on Microsatellite Markers. Int. J. Mol. Sci..

[4] McNeil M., Roberts A.M.I., Cockerell V., Mulholland V. (2004). Real-time PCR assay for quantification of Tilletia caries contamination of UK wheat seed. Plant Pathol..

[5] Tang J., Chen X., Yan Y., Huang J., Luo C., Tom H., Zheng L. (2021). Comprehensive transcriptome profiling reveals abundant long non-coding RNAs associated with development of the rice false smut fungus, Ustilaginoidea virens. Environ. Microbiol..

[6] Schilling L., Matei A., Redkar A., Walbot V., Doehlemann G. (2014). Virulence of the maize smut Ustilago maydis is shaped by organ-specific effectors. Mol. Plant Pathol..

[7] Zhou Y., Qu Y., Zhu M., Liu J., Wang Y., Song H., Feng B. (2016). Genetic diversity and virulence variation of *Sporisorium destruens* isolates and evaluation of broomcorn millet for resistance to head smut. Euphytica.

[8] Zuo W., Ökmen B., Depotter J.R.L., Ebert M.K., Redkar A., Misas Villamil J., Doehlemann G. (2019). Molecular Interactions Between Smut Fungi and Their Host Plants. Annu. Rev. Phytopathol..

[9] Ghareeb H., Becker A., Iven T., Feussner I., Schirawski J. (2011). *Sporisorium reilianum* Infection Changes Inflorescence and Branching Architectures of Maize. Plant Physiol..

[10] Dwivedi S., Upadhyaya H., Senthilvel S., Hash C., Prasad M. (2012). Millets: Genetic and Genomic Resources. Plant Breeding Reviews.

[11] Wu E., Zhang D., Liu J., Liu Y., Gao X., Gao J., Feng B. (2019). Study on responses of leaf protective enzyme and endogenous hormone to smut fungus stress in broomcorn millet. Plant Physiol. J..

[12] Jin F., Liu J., Wu E., Yang P., Gao J., Gao X., Feng B. (2021). Leaf Transcriptome Analysis of Broomcorn Millet Uncovers Key Genes and Pathways in Response to *Sporisorium destruens*. Int. J. Mol. Sci..

[13] Snetselaar K.M., Mims C.W. (1992). Sporidial Fusion and Infection of Maize Seedlings by the Smut Fungus Ustilago maydis. Mycologia.

[14] Zuo W., Chao Q., Zhang N., Ye J., Tan G., Li B., Xing Y., Zhang B., Liu H. (2014). A maize wall-associated kinase confers quantitative resistance to head smut. Nat. Genet..

[15] Oh B.J. (1994). Identification of molecular markers linked to head smut resistance gene (Shs) in sorghum by RFLP and RAPD analyses. Phytopathology.

[16] Li Y., Zou J., Xu X. (2007). Optimization Polyacrylamide Gel Electrophoresis Conditions for Sorghum SSR Molecular Markers. Rain Fed. Crops.

[17] Horst R.J., Doehlemann G., Wahl R., Hofmann J., Schmiedl A., Kahmann R., Kaݶmper J., Sonnewald U., Voll L.M. (2010). Ustilago maydis Infection Strongly Alters Organic Nitrogen Allocation in Maize and Stimulates Productivity of Systemic Source Leaves. Plant Physiol..

[18] Howlett B.J., Doehlemann G., van der Linde K., Aßmann D., Schwammbach D., Hof A., Mohanty A., Jackson D., Kahmann R. (2009). Pep1, a Secreted Effector Protein of Ustilago maydis, Is Required for Successful Invasion of Plant Cells. PLoS Pathog..

[19] Yuanxia L., Guohao Z., Enguo W., Dazhong Z., Long L., Honglu W., Baili F. (2019). Field Identification and Evaluation of Smut Resistant Broomcorn Millet Resources Based on Agronomic Traits. J. Northwest A&F Univ..

[20] Fang Y., Fengxian L., Xiaoqiang S., Haiyan L., Yanrui D., Hai L. (2022). Identification of Smut Disease and Analysis of Related Agronomic Traits in 365 Proso Millet Germplasm Resources. Seed Sci. Technol..

[21] Cheng G., Liu H., Tianwang Z., Chunming W., Lei Z., Xiaojie Z., Kongjun D., Tianyu Y. (2025). Resistance of 32 Broomcorn Millet Germplasms to Head Smut and Occurrence Conditions. Plant Prot..

[22] Hu G.G., Linning R., Bakkeren G. (2002). Sporidial mating and infection process of the smut fungus, Ustilago hordei, in susceptible barley. Can. J. Bot..

[23] Su Y., Wang S., Guo J., Xue B., Xu L., Que Y., Cattori V., Marshall J.C., Tian B. (2013). A TaqMan Real-Time PCR Assay for Detection and Quantification of *Sporisorium scitamineum* in Sugarcane. Sci. World J..

[24] Yan M., Cai E., Zhou J., Chang C., Xi P., Shen W., Li L., Jiang Z. (2016). A Dual-Color Imaging System for Sugarcane Smut Fungus *Sporisorium scitamineum*. Plant Dis..

[25] Göhre V., Spallek T., Häweker H., Mersmann S., Mentzel T., Boller T., de Torres M., Mansfield J.W., Robatzek S. (2008). Plant Pattern-Recognition Receptor FLS2 Is Directed for Degradation by the Bacterial Ubiquitin Ligase AvrPtoB. Curr. Biol..

[26] Thomma B.P.H.J., Nürnberger T., Joosten M.H.A.J. (2011). Of PAMPs and Effectors: The Blurred PTI-ETI Dichotomy. Plant Cell.

[27] Macho A.P., Zipfel C. (2014). Plant PRRs and the Activation of Innate Immune Signaling. Mol. Cell.

[28] Tian W., Hou C., Ren Z., Wang C., Zhao F., Dahlbeck D., Hu S., Zhang L., Niu Q., Li L. (2019). A calmodulin-gated calcium channel links pathogen patterns to plant immunity. Nature.

[29] Wu E., Liu L., Zhu M., Wu H., Yang Q., Li J., Han X., Feng B. (2022). The Life Cycle and Ultrastructure of the Host Response of the Smut Pathogen *Anthracocystis destruens* on Broomcorn Millet. Phytopathology.

[30] Wu E., Li X., Ma Q., Wang H., Han X., Feng B. (2024). Comparative Multi-Omics Analysis of Broomcorn Millet in Response to *Anthracocystis destruens* Infection. Phytopathology.

[31] Fan W., Qi M., Li Z., Zuo Y., Zhao M., Liu H., Wen Y., Wang X., Bian L., Zhang L. (2026). Temporal Metabolic Reprogramming Reveals Stage-Specific Adaptations in Proso Millet Resistance Against Head Smut. Metabolites.

[32] Pertea M., Pertea G.M., Antonescu C.M., Chang T.-C., Mendell J.T., Salzberg S.L. (2015). StringTie enables improved reconstruction of a transcriptome from RNA-seq reads. Nat. Biotechnol..

